# Factors affecting quality of life in adults with HIV: A local cross-sectional study

**DOI:** 10.4102/sajp.v79i1.1921

**Published:** 2023-11-27

**Authors:** Karina Berner, Quinette A. Louw

**Affiliations:** 1 Department of Health and Rehabilitation Sciences, Faculty of Medicine and Health Sciences, Stellenbosch University, Cape Town, South Africa

**Keywords:** comprehensive care, EQ-5D-5L, HIV, patient-reported outcome measures, self-reported health

## Abstract

**Background:**

Understanding health-related quality of life (HRQOL) among people with HIV (PWH) can inform strategies to maintain or improve health and functioning. Most HRQOL research has focused on resource-rich settings, underrepresenting younger cohorts in low-resource settings.

**Objectives:**

To assess HRQOL and associated factors in PWH visiting two primary healthcare clinics in the Western Cape, South Africa.

**Method:**

A cross-sectional study included 48 PWH (58.3% women; mean age: 39.2 [10.3]). Health-related QOL was assessed using EQ-5D-5L descriptive domains, visual analogue scale (EQ-VAS), and index score (EQ-index). Mobility was assessed using clinical tests. Tobit regression determined associations.

**Results:**

Mean and median EQ-VAS scores were 88.14 (16.35) and 95.00. Mean and median EQ-index scores were 0.84 (0.10) and 0.90. PWH reported problems as pain/discomfort (35.4%), depression/anxiety (25.0%), mobility (22.9%), usual activities (18.7%) and self-care (12.5%) domains. Slow chair rise (*p* = 0.012), low income (*p* = 0.030), longer HIV duration (*p* = 0.009) and polypharmacy (*p* = 0.034) were associated with lower HRQOL. Antiretroviral therapy (ART) adherence was associated with higher HRQOL (*p* = 0.020).

**Conclusion:**

Despite high overall HRQOL, specific domains presented challenges to PWH. Health-related QOL was associated with chair rise repetitions, income, HIV duration, polypharmacy, and treatment adherence. Comprehensive care and contextualised interventions to address these through rehabilitation, including health promotion, are proposed strategies for future investigation.

**Clinical implications:**

Clinicians should be cognisant of potential physical and mental functioning problems, and factors related to drug therapy, socio-economic status and disease duration that may affect HRQOL even in seemingly unimpaired PWH.

## Introduction

Advancements and improved roll-out of combination antiretroviral therapy (cART) have delayed mortality and led to human immunodeficiency virus (HIV) becoming a chronic condition. Combination antiretroviral therapy has incontestable benefits for enhancing health and survival. However, the long-term impact that living and ageing with HIV and its clinical manifestations, ongoing drug treatment, and comorbidities has on individuals’ lives, has become a concern (Mokgethi et al. 2022; O’Brien et al. 2020b; Seguiti et al. 2022). Accordingly, as part of the comprehensive care for people with HIV (PWH), prioritising the enhancement of quality of life (QOL) has become essential (Vu et al. 2020). Data from patient-reported outcomes – which have become increasingly important in HIV care decision-making (Kall et al. 2020) – have shown that cART access and viral suppression alone do not holistically meet the needs of PWH, and that additional strategies are necessary to optimise well-being and QOL (Kall et al. 2020). In fact, QOL has been proposed as a ‘fourth 90’ in monitoring the global HIV response under the Joint United Nations Programme on HIV/AIDS (UNAIDS) 90–90–90 targets (Kall et al. 2020). In South Africa, most PWH are of working age (Risher et al. 2021), and chronic management is thus needed at relatively early ages, and potentially for an extended period. This further underscores the importance of reprioritised investigations for understanding the impact that living with HIV has on individuals’ QOL.

Health-related quality of life (HRQOL) narrows the broader aspects of QOL to health-related aspects – that is, how a person’s health status, including the effects of disease and/or treatment, impacts their daily functioning and perceived well-being in the physical, mental, and social domains of life (Raina 2019). Health-related quality of life directly measures an individual’s health status, longevity, and the effects of healthcare utilisation on their QOL (Nigusso & Mavhandu-Mudzusi 2021). Although no single agreed-upon definition exists, HRQOL is agreed as being complex, multidimensional, and concerned with the individual’s perspective (De Wit & Hajos 2023) – as such, emphasising patient-centred care. It has commonly been reported that PWH have lower HRQOL than the general population (Pozniak 2014).

However, most studies investigating HRQOL in PWH have hailed from high-income countries (HICs) and/or were conducted prior to the rollout of cART in sub-Saharan Africa (Haraldstad et al. 2019). Emerging research during the cART era suggests that most domains of PWH’s HRQOL (possibly apart from the mental health domain [Zhou et al. 2021]) have become comparable to that of people with other chronic conditions (Engelhard et al. 2018; Ronel et al. 2018) and/or the general population (Thomas et al. 2017). Nevertheless, as HRQOL particularly relates to the development of service models bridging multidisciplinary boundaries for overall health enhancement (Biraguma, Mutimura & Frantz 2018; Mokgethi et al. 2022), the identification of HRQOL-associated factors, with the goal of optimising PWH’s HRQOL remains of research and clinical interest (Biraguma et al. 2018).

A myriad of sociodemographic, clinical, psychological, and behavioural factors may influence PWH’s HRQOL. A systematic review investigating HRQOL measured using the European Quality of Life Five-Dimensions questionnaire (EQ-5D-5L) across various diseases (Zhou et al. 2021), report (among four studies from lower-middle and upper-middle income countries) that PWH with comorbidities and more progressive disease have lower HRQOL. In meta-analyses including HICs and low- or middle-income countries (LMICs), a lower QOL was related to lower socioeconomic status, stigma, age < 35 years, and CD4 count < 200; and a higher QOL is associated with social support, time of diagnosis, and access to medical services (Ghiasvand et al. 2019a, 2019b). Additional factors for which varying results have been reported across studies from LMICs include gender or sex, age, marital status, educational attainment, employment, income, smoking, alcohol and drug use, physical function (grip strength), physical activity level, hypertension, abdominal obesity, co-morbidities and pill burden, ART use and duration, HIV duration, HIV disease severity or stage and viral load (Ahmed et al. 2021; Biraguma et al. 2018; Dang et al. 2018; Lédo et al. 2018; Louwagie et al. 2007; Maleki et al. 2020; Mokgethi et al. 2022; Nglazi et al. 2014; Tran, Ohinmaa & Nguyen 2012; Thomas et al. 2017). Factors that may be particularly relevant to informing a multidisciplinary management approach, such as non-communicable disease risk factors and lifestyle or behavioural aspects (Biraguma et al. 2018) or functioning, have also become a recent subject of investigation.

Physical function and mobility have only been included in HRQOL research relatively recently and even less so in low resourced settings – and mostly in older, ART-naïve, or neurologically compromised samples (Biraguma et al. 2018; Erlandson et al. 2014; Galantino et al. 2014; Lédo et al. 2018). Findings in these studies suggest that PWH with impaired mobility function may also have worse QOL. This is of particular concern considering that in South Africa, at least 70% of adults seeking primary care have one or more functioning problem(s) and HIV counts among the top 10 diseases contributing to disability in the country (Charumbira, Berner & Louw 2022).

Investigations into PWH’s QOL have become increasingly important since cART was first introduced, yet most HRQOL research is not conducted in LMICs, which carry the heaviest HIV burden (Haraldstad et al. 2019; Vu et al. 2020). The relatively younger profile of PWH in Africa (new cases primarily occurring among young adults) (Risher et al. 2021), along with concerns of accelerated ageing (Erlandson, Guaraldi & Falutz 2016), implies that subtle impairments may occur that could impact HRQOL in at younger-than-expected ages in the short or longer term. Such individuals might not be routinely considered as being at risk of impending HRQOL-related problems. Further research is thus required to gain a better understanding of the influences of sociodemographic, clinical, lifestyle, and functional factors on the HRQOL of PWH residing in low resourced settings – especially in non-elderly cohorts that are not obviously impaired – and including factors that have not (traditionally) been widely researched such as physical function. This may ultimately inform early, targeted, and contextually relevant prevention and/or intervention strategies aimed at maintaining and/or improving HRQOL in these settings (Biraguma et al. 2018). The aim of our study was to assess HRQOL in PWH, without obvious risk factors for locomotor impairment, visiting two primary healthcare clinics in the Western Cape of South Africa, and determine associations between sociodemographic, lifestyle, and functional characteristics with HRQOL.

## Methods

This cross-sectional descriptive study, used data from a large study that assessed gait biomechanics, function, and falls in people with and without HIV. The primary outcomes of the large study, which included self-reported function as assessed using three EQ-5D-5L domains, are reported elsewhere (Berner et al. 2021). The full HRQOL results, assessed using the EQ-5D-5L, for PWH with available data are reported here. Our study was conducted in the Cape Winelands district (Breede Valley sub-district) of the Western Cape, South Africa. The Breede Valley has an unemployment rate of 11.8% and its GDP per capita is well below the average rates for the Western Cape (Western Cape Government 2017).

The area has seen significant growth in informal or peri-urban areas over recent years (Cullis et al. 2018). Income inequality levels increased between 2010 and 2016, with a Gini coefficient marginally lower (0.58) than in the Western Cape (0.61) and South Africa (0.63), but well above the global average of 38.8.

The participants included adults who reside in the Cape Winelands district and attend two public primary care Community Health Centres in the Breede Valley sub-district. These clinics were conveniently selected because of existing networks and permission for HIV-related research from the provincial department of health. Participants were consecutively evaluated for participation between June 2016 and December 2017. As patients arrived randomly at the clinic for care, we did not expect consecutive sampling to introduce any sampling biases with respect to sex, age, socio-demographics, and health status.

Study eligibility criteria are related to the large gait-focused study (Berner et al. 2021), which aimed to include adults without obvious predisposing factors to mobility function impairments. Adults aged 18–65, with a body mass index (BMI) < 25 kg/m^2^ (as obesity may have significant impacts on locomotor function in PWH [Bauer, Wu & Wolfson 2011]), and who were independently ambulatory were included. Exclusion criteria were pregnancy or within the first 3 months after giving birth, acute opportunistic infection, peripheral neuropathy (PN) (given likely significant impacts on locomotor function [Erlandson et al. 2019]), major neurological conditions or neuromusculoskeletal impairments and/or injury affecting walking gait, severe visual impairment, or acute alcohol intake (because of impact on locomotor performance [Ando et al. 2008]). Peripheral neuropathy screening was based on folder information and/or self-report via Single Question Neuropathy Screening (‘yes’ to any of three symptoms [Cherry et al. 2005; Cettomai et al. 2013]). This was augmented by a standardised brief neurological conduction battery (Cettomai et al. 2010, 2013) consisting of assessing ankle deep tendon reflex (Cettomai et al. 2010, 2013) in sitting, using a queen’s square hammer, and light touch sensation with a cotton ball (Stolk-Hornsveld et al. 2006) by experienced physiotherapists (first author [K.B.] or research assistant). Folder or self-report confirmation, or presence of ≥ 1 PN sign (reduced and/or absent light touch sensation or ankle reflex [Grade 1 or 0]) was considered indicative of PN (Cettomai et al. 2010, 2013). Acute alcohol intake was assessed using a single dichotomous 24-h recall question based on ingestion on the day of testing (Agarwal, Fulgoni & Lieberman 2016).

All PWH who participated in the large study and had valid HRQOL data (*n* = 48), were included in our present analysis. Based on α = 0.05 and an EQ-VAS mean score difference of 4.54 in a South African study among PWH (Narsai et al. 2016), the power of regression analysis to detect a significant finding (relationship different from 0) was calculated as > 95% for the sample size of 48, and therefore adequate.

### Data sources and measures

Single-visit assessments were conducted at the clinics or a dedicated adjacent venue. Participants completed demographic and HRQOL questionnaires at the start of the session, before performing functional performance tests. The main languages in the Cape Winelands district are Afrikaans, English, and isiXhosa. For the HRQOL questionnaire, participants indicated which language they were comfortable in, and the corresponding standard translated version of questionnaire was provided. The physical testing procedures were conducted in a randomised order and interspersed with 5-min rest periods.

### Variables

#### Health-related quality of life (dependent variable)

Health-related quality of life was assessed using the EQ-5D-5L, a standardised and extensively validated self-report questionnaire. The instrument provides a five-dimensional profile of HRQOL using the EQ-5D descriptive system, subjective overall health via the EuroQol Visual Analogue Scale (EQ-VAS) and a utility-based index of health status (EQ-index). The EQ-5D-5L is available in South African English, Afrikaans, and isiXhosa and although not specifically designed for HIV infection, has been validated, tested for reliability, and successfully applied in PWH (Cronbach’s alpha 0.85 [Louwagie et al. 2007; Tran et al. 2012]), including in South Africa (Mkoka et al. 2003; Jelsma et al. 2004; Louwagie et al. 2007). We used the EQ-5D-5L because of its brevity, suitability for studies in PWH, and availability in local languages (South African English, isiXhosa and Afrikaans) (Mkoka et al. 2003; Jelsma et al. 2004; Louwagie et al. 2007). The tool was pilot-tested on five PWH to ensure understanding of the questions in the sampled community.

The EQ-5D descriptive system comprises five dimensions (mobility, self-care, usual activities, pain/discomfort, and anxiety or depression), with each dimension offering five response options that correspond to the severity level (1 = no problems, to 5 = extreme problems). The combination of each level on each dimension enables the definition of 3125 ‘health states’, between ‘11111’ (‘best imaginable health’) to ‘55555’ (‘worst imaginable health’).

The EQ-VAS is a 20-cm visual analogue interval scale to gauge an individual’s self-reported overall perception of health on the day of assessment. The individual’s self-rated health is plotted on a vertical scale that spans from 0 (representing the poorest possible health) to 100 (highest possible health perception).

The EQ-index is a weighted health index value based on societal preference weights for different health states. A score is obtained by converting the descriptive system health state into a single value (utility index score) and applying country-specific valuation weights derived from studies in the general population (EuroQol Research Foundation 2019). Health state preferences can differ between countries/regions as they are commonly representative of national or regional values (EuroQol Research Foundation 2019). Where a standard EQ-5D-5L value set does not exist for a specific country, a value set from a country that bears the closest resemblance to the country in question can be selected (EuroQol Research Foundation 2019). As no South African valuation algorithms currently exist, we used value sets developed for Zimbabwe (a low-income country in Southern Africa) to derive index scores (EuroQol Research Foundation 2019; Kastien-Hilka et al. 2017). The EQ index scores range from < 0 (i.e., worse than death; 0 being representative of a health state equivalent to death) to 1 (representing full health) (EuroQol Research Foundation 2019).

The EQ-VAS together with the descriptive system is useful for gaining an aggregate impression of an individual’s health status (specific aspects and overall). It may be particularly relevant to clinical decision making, where the patient perspective is important, as it presents the individual’s own assessment of health status (EuroQol Research Foundation 2019). The EQ-index, as a societal valuation of the person’s health status, may in contrast be a more useful measure to inform economic assessments of healthcare interventions where the societal perspective is preferred (e.g., it is often used to calculate quality-adjusted life years [QALYs]) (EuroQol Research Foundation 2019).

#### Independent variables

Sociodemographic, lifestyle-related, and clinical information was extracted from the large study. The following sociodemographic and lifestyle information was collected: age, sex, education level (less than Grade 12 versus Grade 12 and above), employment status (employed versus unemployed), monthly household income (four categories from < R1000 to > R20,000), tobacco smoking (ever vs. never smoked), and regular physical activity (yes vs. no). Clinical information (from patient folder information, self-report and blood tests) included multimorbidity (presence of ≥ 2 chronic conditions, including HIV [The Academy of Medical Sciences 2018]), polypharmacy (intake of ≥ 5 medications, including ART [Danjuma et al. 2022]), time since HIV diagnosis (four categories from < 2 years to > 15 years), current CD4+ count (three categories of < 200, 200–500 or > 500 cells/mm^3^), viral load (detectable vs. undetectable), current ART use (yes vs. no), ART duration (three categories), and ART adherence (two categories: taking ART as prescribed all or most of the time, vs not compliant) (Rudy et al. 2009).

Mobility function was assessed using physical performance tests, performed after EQ-5D-5L questionnaire completion (to avoid performance influencing subjective responses). These assessments were performed by two Health Professions Council of South Africa (HPCSA)-registered physiotherapists, experienced in the assessment of human movement, and in the process of completing postgraduate degrees (an MSc and PhD, respectively). The physical performance tests included the Health ABC Physical Performance Battery (PPB) (total score), 6-m walk test (6mWT) (usual-paced and fast speed in m/s), five-times sit-to-stand test (5STS) (seconds to complete), and 30-second chair stand test (30CST) (number of repetitions). Full details regarding the physical performance tests are reported elsewhere (Berner et al. 2021).

Briefly, the PPB assesses lower limb function and consists of four sub-tests that mimic daily activities: standing balance, five chair rises, 6-m usual-paced gait speed, and a narrow-walk test of dynamic balance. Physical Performance Battery total score ranges continuously from 0 to 4, with higher scores indicating better performance. Given the lack of cut-off score guidelines for the PPB, poor test performance was defined as a result of ≥ 2 standard deviations (s.d.) (Richert et al. 2011) from the mean performance of HIV-negative participants in the large study (Berner et al. 2021).

To measure short distance gait speed, the participant was requested to walk on a straight 6-m course at a comfortable pace (usual-paced gait speed) or as fast as they could (fast gait speed). Although there are published cut-points for what generally constitutes ‘slow’ gait, gait speed strongly relates to the community that a person hails from (Ebersbach et al. 2000). Thus, poor performance (slow gait) was defined as a result that was slower by ≥ 2 s.d. from the mean gait speed in HIV-negative participants in the large study (Berner et al. 2021; Richert et al. 2011).

Chair rise tests are measures of functional mobility and take less time to administer than even short performance batteries. These tests are usually employed to evaluate older adults; however, they are also valid as measurements of physical performance in healthy younger adults (Gurses et al. 2018).

Although the 5STS test and 30CST require execution of the same motion, they are not considered interchangeable as the 5STS test is an indication of lower limb power, speed, and dynamic balance, whereas the 30CST test is a proxy of lower limb endurance and muscle strength. Results were interpreted based on published data from the general population, using sex- and/or age-specific performance values, where available (Richert et al. 2011). Poor performance was defined by a result of > 2 s.d. from the expected sex- and/or age-specific mean in the general population (Bohannon et al. 2010; McKay et al. 2017).

### Data analysis

Statistical analyses were conducted using Statistical Package for Social Sciences (SPSS) version 27 and STATA V.14.2. Statistical significance was set at 5%. For continuous or numerical data, normality was checked using the Shapiro–Wilk Test (given the sample size of < 50).

Socio-demographic, lifestyle, and clinical participant characteristics were described using frequencies (*n*, %), central tendency (mean, median), and spread (minimum and maximum, 95% confidence interval [CI]). Health-related quality of life results were entered into an Microsoft Excel sheet and scored as per the EQ-5D-5L´s user manual (EuroQol Research Foundation 2019). Summary statistics were derived, including frequencies and percentages for the five EQ-5D dimensions. EuroQol Visual Analogue Scale results were presented as a continuous score of subjective overall health. The EQ-5D calculator (Van Hout et al. 2012) was used to calculate EQ-index scores according to Zimbabwean valuation algorithms (EuroQol Research Foundation 2019; Kastien-Hilka et al. 2017). The EQ-VAS and -index scores were summarised using mean, s.d., 95% CI, median, interquartile range (IQR), minimum, and maximum values.

Mann–Whitney U and Kruskal–Wallis tests were used to examine differences in EQ-index and -VAS scores between PWH with different characteristics. Subsequently, the impact of the variables on PWH’s HRQOL was assessed using Tobit regression models (upper censoring at 1 for EQ index scores or 100 for the EQ-VAS). The models included all the statistically significant independent variables (*p* < 0.05) from the previous tests. Censored regression models are used to estimate the linear relationship between variables when the dependent variable has left or right censoring, as is often the case in HRQOL measures (Tran et al. 2012).

### Ethical considerations

Study approval was obtained from the Stellenbosch University Health Research Ethics Committee (N15/05/043) and the Western Cape Department of Health. To ensure participant confidentiality, identifying information was not recorded on data documents and study codes were assigned instead. A separate linking document was locked in an access-restricted location. Written informed consent, which included specific consent for HIV testing, was obtained from all participants. Pre- and post-test counselling was available as necessary. Written permission was obtained from the EuroQol Research Foundation for using the South African English, Afrikaans, and isiXhosa versions of the EQ-5D-5L instrument.

## Results

### Participants and sample characteristics

**FIGURE 1:**
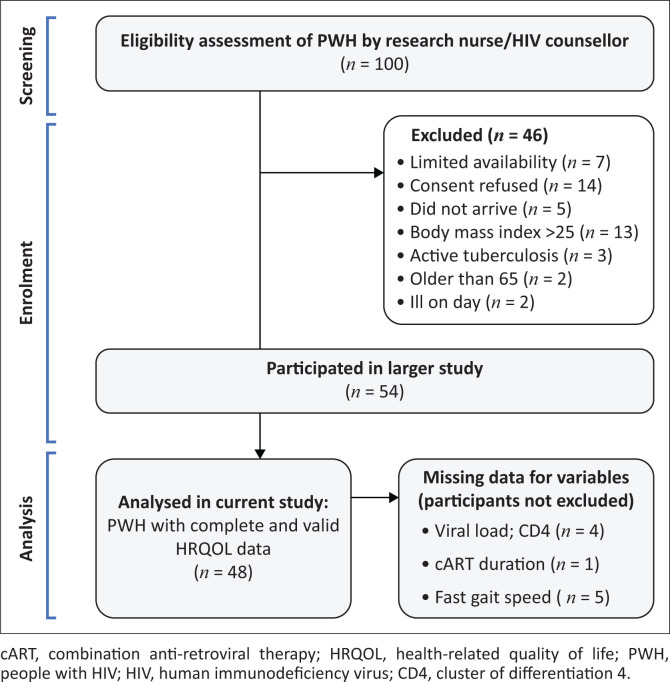
Selection of participants for the large study and subsequent inclusion in our health-related quality of life analysis.

Out of the 54 PWH who participated in the large study, 48 had complete and correct EQ-5D-5L data (Berner et al. 2021) and were included in the analysis. Figure 1 depicts screening and selection of PWH for the large study and subsequent inclusion of *n* = 48 participants in the HRQOL analysis reported here.

The mean age of participants was 39.2 (10.3) years, and more than half were women (58.3%). Most participants (45.8%) had an HIV duration of 5–15 years. Most (89.6%) were using cART (76.2% for > 5 years), although 23.3% of cART-users reported non-adherence. Just under half (47.7%) had undetectable viral loads. Mean (s.d.) cluster of differentiation 45 (CD4+) count was 443.61 (233.84) (95% CI: 375.56–511.67) cells/mm^3^, ranging from 49 cells/mm^3^ to 922 cells/mm^3^ (Table 1).

**TABLE 1:** Sample characteristics (*n* = 48).

Characteristic	*n*	*N*	%	Mean	s.d.	95% CI	Mdn	IQR	Range
Age in years	48	48	100.0	39.2	10.3	36.2–42.1	37.0	14.5	20.5–64.2
**Age group (years)**									
18–49	38	48	79.2	-	-	-	-	-	-
50+	10	48	20.8	-	-	-	-	-	-
Women	28	48	58.3	-	-	-	-	-	-
Education < Grade 12	27	48	56.3	-	-	-	-	-	-
Employed	29	48	60.4	-	-	-	-	-	-
**Monthly household income**				-	-	-	-	-	-
< R1000.00	16	48	33.3	-	-	-	-	-	-
R1000.00 – R4999.00	21	48	43.8	-	-	-	-	-	-
R5000.00 – R9999.00	8	48	16.7	-	-	-	-	-	-
R10 000.00 – R20 000.00	3	48	6.3	-	-	-	-	-	-
> R20 000.00	0	48	0.0	-	-	-	-	-	-
Ever smoked (current or ex-smoker)	34	48	70.8	-	-	-	-	-	-
Multimorbidity	16	48	33.3	-	-	-	-	-	-
Polypharmacy	6	48	12.5	-	-	-	-	-	-
Physically active	42	48	87.5	-	-	-	-	-	-
PPB score	48	48	100.0	2.39	0.43	2.27–2.52	2.47	0.59	1.25–3.01
6mWT usual (m/s)	48	48	100.0	1.01	0.25	0.94–1.08	1.02	0.38	0.47–1.51
6mWT fast (m/s)§	43	48	89.6	1.53	0.35	1.43–1.08	1.49	0.55	0.87–2.35
5STS (sec)	48	48	100.0	11.60	2.89	10.76–12.44	11.21	4.33	6.70–18.00
30CST (reps)	48	48	100.0	16.00	5.00	15.00–18.00	15.00	9.00	8.00–30.00
Poor PPB	14	48	29.2	-	-	-	-	-	-
Poor 6mWT usual	13	48	27.1	-	-	-	-	-	-
Poor 6mWT fast	7	43	16.3	-	-	-	-	-	-
Poor 5STS	36	48	75.0	-	-	-	-	-	-
Poor 30CST	8	48	16.7	-	-	-	-	-	-
**Time since HIV diagnosis (years)**				-	-	-	-	-	-
< 2	9	48	18.8	-	-	-	-	-	-
2–5	14	48	29.2	-	-	-	-	-	-
> 5–15	22	48	45.8	-	-	-	-	-	-
> 15	3	48	6.3	-	-	-	-	-	-
**CD4+ count†**				-	-	-	-	-	-
< 200 cells/mm^3^	6	44	13.6	-	-	-	-	-	-
200 cells/mm^3^ – 500 cells/mm^3^	21	44	47.7	-	-	-	-	-	-
> 500 cells/mm^3^	17	44	38.6	-	-	-	-	-	-
**Viral load†**				-	-	-	-	-	-
Detectable (≥ 50 cp/mL)	23	44	52.3	-	-	-	-	-	-
On ART	43	48	89.6	-	-	-	-	-	-
**ART duration (years)‡**				-	-	-	-	-	-
< 2	5	42	11.9	-	-	-	-	-	-
2–5	5	42	11.9	-	-	-	-	-	-
> 5	32	42	76.2	-	-	-	-	-	-
ART adherent	33	43	76.7	-	-	-	-	-	-

Note: Multimorbidity was defined as the presence of ≥ 2 chronic conditions (including HIV) (The Academy of Medical Sciences 2018). Polypharmacy was defined as the intake of ≥ 5 medications (including ART) (Danjuma et al. 2022).

30CST, 30-second chair stand test; 5STS, 5-times sit-to-stand test; 6mWT, 6-m walk test; ART, antiretroviral therapy; s.d., standard deviation; IQR, interquartile range; Mdn, median; PPB, physical performance battery; HIV, human immunodeficiency virus; CD4, cluster of differentiation 4.

† , No CD4 and viral load data available for *n* = 4 participants who had no laboratory results or values recorded in medical records.

‡ , ART duration information not available for *n* = 1 participant who did not know.

§ , Fast gait results missing for *n* = 5 participants.

### Health-related quality of life results

Participants had a mean EQ-VAS score of 88.14 (16.35) and a median of 95.00. Participants had a mean EQ-index score of 0.84 (0.10), and a median of 0.90. The EQ-VAS and -index scores were skewed right, that is, towards the higher values. The most reported problems were in the pain or discomfort domain, where 35.4% of participants reported having problems (i.e., all levels inclusive). This was followed by problems in the depression or anxiety domain (25.0%), the mobility domain (22.9%), the usual activities domain (18.7%), and the self-care domain (12.5%). A total of 26/48 (54.2%) of participants reported no problems in any domains (i.e., a full health state of 11111) (Table 2).

**TABLE 2:** Health-related quality of life of people with HIV.

EQ-5D-5L scores and dimensions†	*n*	*N*	%	Mean	s.d.	95% CI	Mdn	IQR	Range
EQ-VAS score	48	48	100.0	88.14	16.35	83.39–92.88	95.00	18.75	50.00–100.00
EQ-index score	48	48	100.0	0.84	0.10	0.81–0.87	0.90	0.10	0.48–0.90
**EQ-5D Mobility**	-	-	-	1.27	0.56	1.12–1.43	1.00	0.00	1.00–3.00
1 – No problems	37	48	77.1	-	-	-	-	-	-
2 – Slight problems	9	48	18.8	-	-	-	-	-	-
3 – Moderate problems	2	48	4.2	-	-	-	-	-	-
4 – Severe problems	0	48	0.0	-	-	-	-	-	-
5 – Extreme problems	0	48	0.0	-	-	-	-	-	-
**EQ-5D self-care**	-		-	1.21	0.62	1.03–1.39	1.00	0.00	1.00–4.00
1 – No problems	42	48	87.5	-	-	-	-	-	-
2 – Slight problems	3	48	6.3	-	-	-	-	-	-
3 – Moderate problems	2	48	4.2	-	-	-	-	-	-
4 – Severe problems	1	48	2.1	-	-	-	-	-	-
5 – Extreme problems	0	48	0.0	-	-	-	-	-	-
**EQ-5D usual activities**	-	-	-	1.27	0.64	1.08–1.46	1.00	0.00	1.00–4.00
1 – No problems	39	48	81.3	-	-	-	-	-	-
2 – Slight problems	6	48	12.5	-	-	-	-	-	-
3 – Moderate problems	2	48	4.2	-	-	-	-	-	-
4 – Severe problems	1	48	2.1	-	-	-	-	-	-
5 – Extreme problems	0	48	0.0	-	-	-	-	-	-
**EQ-5D pain/discomfort**	-		-	1.52	0.80	1.29–1.75	1.00	1.00	1.00–4.00
1 – No problems	31	48	64.6	-	-	-	-	-	-
2 – Slight problems	10	48	20.8	-	-	-	-	-	-
3 – Moderate problems	6	48	12.5	-	-	-	-	-	-
4 – Severe problems	1	48	2.1	-	-	-	-	-	-
5 – Extreme problems	0	48	0.0	-	-	-	-	-	-
**EQ-5D depression/anxiety**	-	-	-	1.38	0.76	1.15–1.60	1.00	1.00	1.00–4.00
1 – No problems	36	48	75.0	-	-	-	-	-	-
2 – Slight problems	8	48	16.7	-	-	-	-	-	-
3 – Moderate problems	2	48	4.2	-	-	-	-	-	-
4 – Severe problems	2	48	4.2	-	-	-	-	-	-
5 – Extreme problems	0	48	0.0	-	-	-	-	-	-

Note: EQ-5D-5L VAS: 0 (worst) to 100 (best health); EQ-5D-5L index: 0 (worst) to 1 (best HRQOL).

EQ-5D-5L, European Quality of Life Five-Dimensions questionnaire; EQ-index, EuroQoL index of health status; EQ-VAS, EuroQoL Visual Analogue Scale; HIV, human immunodeficiency virus; PWH, people with HIV; QoL, quality of life; s.d., standard deviation; Mdn, median; IQR, interquartile range; HRQoL, health-related quality of life.

† , Showing severity-level response options per dimension.

### Factors associated with health-related quality of life

For the EQ-VAS, results from initial analyses indicated significantly higher scores (better self-perceived overall health) in those with the highest category of total monthly household income (*p* = 0.038), and significantly lower scores (poorer self-perceived health) in those with poor 30CST performance (*p* < 0.001), slow 6mW speed (*p* = 0.011), those who were on cART (*p* = 0.046), and those who were cART non-adherent (*p* = 0.002). For the EQ-index, lower scores (worse HRQOL/community-preferred health state) were observed for PWH with multimorbidity (*p* = 0.003), polypharmacy (*p* = 0.014), and those with a HIV duration of > 15 years (*p* = 0.017) (Table 3).

**TABLE 3:** Results from initial Mann–Whitney U and Kruskal–Wallis analyses: EuroQol (EQ)-index and -VAS scores for people with HIV (PWH, *n* = 48) according to background and functional categories.

Characteristic	*n*	EQ-Index Mean	s.d.	EQ-Index Mdn	Range	*p*	EQ-VAS Mean	s.d.	EQ-VAS Mdn	Range	*p*
**Gender/sex**						0.470					0.839
Men	20	0.83	0.11	0.90	0.48–0.90	-	91.15	11.95	95.00	50.00–100.00	-
Women	28	0.85	0.09	0.90	0.61–0.90	-	85.98	18.80	95.00	50.00–100.00	-
**Age**						0.507					0.067
18–49	38	0.85	0.08	0.90	0.61–0.90	-	90.41	14.84	95.00	50.00–100.00	-
50+	10	0.81	0.15	0.88	0.48–0.90	-	79.50	19.64	82.50	50.00–100.00	-
**Level of education**						0.916					0.197
Lower than Grade 12	27	0.85	0.08	0.90	0.61–0.90	-	80.98	19.18	90.00	50.00–100.00	-
Grade 12 and higher	21	0.83	0.11	0.90	0.48–0.90	-	93.48	9.84	95.00	60.00–100.00	-
**Total monthly household income**						0.528					0.038*
< R1000.00	16	0.84	0.08	0.87	0.64–0.90	-	89.88	16.96	99.00	50.00–100.00	-
R1000.00 – R4999.00	21	0.84	0.10	0.90	0.61–0.90	-	82.98	18.27	90.00	50.00–100.00	-
R5000.00 – R20 000.00	11	0.85	0.13	0.90	0.48–0.90	-	95.45	6.11	95.00	80.00–100.00	-
> R20 000.00	0	-	-	-	-	-	-	-	-	-	
**Employment status**						0.201					0.879
Employed	29	0.85	0.10	0.90	0.48–0.90	-	88.71	15.26	95.00	51.00–100.00	-
Unemployed	19	0.82	0.10	0.90	0.61–0.90	-	87.26	18.29	95.00	50.00–100.00	-
**Ever smoked**						0.355					0.964
Yes	34	0.83	0.11	0.90	0.48–0.90	-	87.43	17.15	95.00	50.00–100.00	-
No	14	0.86	0.07	0.90	0.70–0.90	-	89.86	14.66	95.00	53.00–100.00	-
**Multimorbidity**						0.003*					0.565
Yes	16	0.78	0.13	0.83	0.48–0.90	-	88.81	14.56	92.5	50.00–100.00	-
No	32	0.87	0.06	0.90	0.70–0.90	-	87.80	17.39	95.00	50.00–100.00	-
**Polypharmacy**						0.014*					1.000
Yes	6	0.73	0.13	0.72	0.61–0.90	-	88.00	19.44	96.50	50.00–100.00	-
No	42	0.86	0.08	0.90	0.48–0.90	-	88.15	16.13	95.00	50.00–100.00	-
**Physically active**						0.078					0.843
Yes	42	0.83	0.10	0.90	0.48–0.90	-	88.62	16.03	95.00	50.00–100.00	-
No	6	0.90	0.00	0.90	0.90–0.90	-	86.43	17.73	95.00	50.00–100.00	-
**5STS time**						0.275					0.098
Normal	12	0.80	0.14	0.87	0.48–0.90	-	94.42	11.30	99.00	60.00–100.00	-
Poor	36	0.86	0.08	0.90	0.61–0.90	-	86.04	17.34	95.00	50.00–100.00	-
**30CST repetitions**						0.938					0.002*
Normal	40	0.84	0.10	0.90	0.48–0.90	-	91.19	14.46	96.50	50.00–100.00	-
Poor	8	0.87	0.05	0.88	0.77–0.90	-	72.88	17.62	80.00	50.00–95.00	-
**6mWT usual speed**						0.743					0.011*
Normal	35	0.84	0.10	0.90	0.48–0.90	-	92.69	11.27	95.00	53.00–100.00	-
Slow	13	0.85	0.10	0.90	0.61–0.90	-	75.88	21.54	80.00	50.00–100.00	-
**6mWT fast speed**						0.987					0.054
Normal	36	0.86	0.08	0.90	0.61–0.90	-	91.42	13.37	95.00	50.00–100.00	-
Slow	7	0.87	0.05	0.90	0.77–0.90	-	72.36	20.12	80.00	50.00–100.00	-
**Health ABC PPB**						0.572					0.116
Normal	34	0.84	0.10	0.90	0.48–0.90	-	91.59	13.36	95.00	50.00–100.00	-
Poor	14	0.86	0.08	0.90	0.61–1.00	-	79.75	20.17	85.00	50.00–100.00	-
**Time since HIV diagnosis (years)**						0.017*					0.388
< 2	9	0.86	0.06	0.90	0.76–0.90	-	96.67	3.54	95.00	90.00–100.00	-
2–5	14	0.82	0.10	0.87	0.61–0.90	-	83.43	20.40	92.50	50.00–100.00	-
> 5	22	0.87	0.07	0.90	0.61–0.90	-	87.84	16.85	95.00	50.00–100.00	-
> 15	3	0.68	0.18	0.77	0.48–0.80	-	86.67	7.64	85.00	80.00–95.00	-
**Detectable viral load**						0.296					0.910
Yes	23	0.85	0.08	0.90	0.61–0.90	-	88.17	15.33	95.00	50.00–100.00	-
No	21	0.83	0.11	0.90	0.48–0.90	-	88.21	16.61	95.00	50.00–100.00	-
**CD4 count (cells/mm^3^)**						0.871					0.262
CD4 < 200	6	0.83	0.10	0.88	0.64–0.90	-	79.17	21.31	87.50	50.00–100.00	-
CD4 200–500	21	0.85	0.08	0.90	0.61–0.90	-	90.36	13.47	95.00	50.00–100.00	-
CD4 count > 500	17	0.84	0.11	0.90	0.48–0.90	-	90.53	15.48	97.50	51.50–100.00	-
**On cART**						0.555					0.046*
Yes	43	0.84	0.10	0.90	0.48–0.90	-	86.87	16.82	95.00	50.00–100.00	-
No	5	0.86	0.09	0.90	0.70–0.90	-	0.99	2.24	100.00	95.00–100.00	-
**cART adherent**						0.166					0.002*
Yes	33	0.83	0.10	0.90	0.48–0.90	-	91.33	13.00	95.00	53.00–100.00	-
No	10	0.87	0.09	0.90	0.61–0.90	-	71.15	20.13	77.50	50.00–100.00	-

30CST, 30-second chair stand test; 5STS, 5-times sit-to-stand test; 6mWT, 6-m walk test; EQ-index, European quality of life index of health status; cART, combination antiretroviral therapy; HIV, human immunodeficiency virus; IQR, interquartile range; Mdn, median; PPB, Physical Performance Battery; VAS, visual analogue scale; s.d., standard deviation; CD4, cluster of differentiation 4.

* , *p* < 0.05.

Final multivariable analyses showed that relatively lower (but not the lowest) monthly household income of R1000.00 – R4999.00, and poor 30CST performance, were retained as being significantly associated with lower EQ-VAS scores (*p* = 0.030 and *p* = 0.012, respectively), while being cART adherent was associated with a better EQ-VAS score (*p* = 0.020) (Table 4). A longer time since HIV diagnosis (> 15 years ago), and polypharmacy, remained significantly associated with lower EQ-index scores (*p* = 0.009 and *p* = 0.034, respectively) (Table 4).

**TABLE 4:** Results of final multivariable Tobit regression models on EuroQol (EQ)-VAS and -index scores.

Variables	Regression coefficient	95% CI	*p*
**EQ-VAS**			
Total household income per month(ref ≤ R1000.00)			
R1000.00 – R4999.00	−15.594	−28.006–3.183	0.015*
R5000.00 – R20 000.00	−3.334	−18.879–12.211	0.667
30CST repetitions (ref = normal)			
Poor	−18.626	−32.615–4.637	0.010*
6mWT speed (usual paced)(ref = normal)			
Slow	−5.350	−18.888–8.188	0.429
cART adherent (ref = no)			
Yes	17.698	4.685–30.712	0.009*
**EQ-INDEX**			
Time since HIV diagnosis(ref ≤ 2 years)			
2–5	−0.024	−0.090–0.042	0.464
> 5	0.014	−0.045–0.073	0.640
> 15	−0.138	−0.241–0.036	0.009*
Multimorbidity (ref = no)			
Yes	−0.046	−0.100–0.006	0.081
Polypharmacy (ref = no)			
Yes	−0.077	−0.148–0.006	0.034*

Note: cART use omitted from model because of collinearity.

30CST, 30-second chair stand test; 6mWT, 6-m walk test; EQ-index, EuroQoL index of health status; EQ-VAS, EuroQoL Visual Analogue Scale; cART, combination antiretroviral therapy; CI, confidence interval; HIV, human immunodeficiency virus; VAS, visual analogue scale.

* , *p* < 0.05.

## Discussion

The assessment of HRQOL is gaining attention worldwide to understand the impact of health problems on individuals’ daily lives. This is particularly important in LMICs, where the burden of chronic diseases such as HIV is disproportionately high, and where the affected population may be younger (Risher et al. 2021). We aimed to assess HRQOL and its associated factors among a relatively young, seemingly unimpaired cohort of PWH from two clinics located in the Western Cape of South Africa. Our findings indicate that although PWH scored high on the HRQOL index and VAS measures, some experienced difficulties with pain or discomfort, depression or anxiety, mobility, usual activities, and self-care. Number of sit-to-stand repetitions in 30 seconds, household income, HIV duration, polypharmacy, and cART adherence were associated with HRQOL.

At face value, our results suggest that the HRQOL of PWH is high, which may not be surprising given the nature of the sample eligibility criteria. This is however also similar to studies including wider profiles of PWH in the modern cART era (Pozniak 2014), including in LMICs (Anosike, Anene-Okeke & Akunne 2021; Nglazi et al. 2014; Thomas et al. 2017). Studies among South African PWH that were conducted after the national rollout of cART report average EQ-index scores between 0.80 and 0.92 (Gow, George & Govender 2013; Louwagie et al. 2007; Thomas et al. 2017), and average EQ-VAS scores between 76.1 and 90.0 (Jelsma et al. 2005; Gow et al. 2013; Nglazi et al. 2014). Furthermore, when comparing PWH’s scores with that of other populations, the HRQOL of PWH seems to have become comparable (Engelhard et al. 2018; Ronel et al. 2018; Thomas et al. 2017;), or better (Gow et al. 2013; Narsai et al. 2016; Seguiti et al. 2022), to that of others living with chronic conditions, in similar low socio-economic circumstances and/or the general population. However, comparing HRQOL scores between populations is complex because of the use of different assessment tools (e.g., differences in PWH and HIV-negative peers’ HRQOL scores may depend on the test used [Gow et al. 2013]), increasingly prevalent multimorbidity in the general population (currently affecting up to one in five South Africans and negatively impacting HRQOL [Roomaney et al. 2022]), and societal inequalities. In terms of the latter, unemployment, poverty, and stressful circumstances have detrimental effects on the HRQOL of South Africans from low-resourced communities regardless of HIV status (Narsai et al. 2016), while better healthcare access and cART infrastructure benefits could in turn contribute to PWH reporting better HRQOL compared with the general community (Martin, Russell & Seeley 2014). Considering the contentious nature of what defines HRQOL and that it is highly subjective, summary scores from quantitative HRQOL tools may obscure nuances that may need further investigation.

Despite high overall HRQOL scores, specific HRQOL domains presented challenges to some PWH (particularly pain or discomfort, depression or anxiety, and mobility). A Capetonian study (Nglazi et al. 2014) found similar proportions of PWH reporting problems related to pain or discomfort (24.7%), anxiety or depression (13.4%), and mobility (6.5%), regardless of cART use and despite high EQ-VAS scores. Although the proportion of PWH reporting problems may be considered small, these results could imply a notable problem at the population level, which could worsen over time. We observed these problematic domains in a relatively young sample without obvious impairment, suggesting a potential increase in disability or the development of new issues in the future. One of the first qualitative studies to investigate the QOL change beyond 1 year after ART initiation, conducted in South African PWH in the public sector, suggests that changes in QOL and associated problems continue to occur in the longer term (Hanass-Hancock et al. 2015). Our findings furthermore echo a recent scoping review, which identified problems related to mobility, pain, and mental health as the most common functioning problems contributing to disability in South African adults across various health conditions (including HIV) (Charumbira, Berner & Louw 2022). These findings may have significant economic and social implications, given the increasing longevity of PWH because of advanced care. The profile reported here begs the question whether the care that PWH are receiving is comprehensive enough to address problems and impairments that could contribute to HRQOL now or in future.

Adherence to cART, rather than use, was associated with better HRQOL. This observation agrees with findings from meta-analyses that the impact of cART use on PWH’s HRQOL may be fairly neutral (Ghiasvand et al. 2019b); suggesting that monitoring adherence may be a better marker of PWHs’ HRQOL. Research into cART adherence has highlighted that the factors linked to non-adherence vary based on region, underlining the necessity for contextualising non-adherence profiles (Heestermans et al. 2016; Ware et al. 2009). In resource-constrained contexts, cART non-adherence has been associated with worse HRQOL, but also with factors such as functional limitations, lack of physical activity, and chronic pain (Dang et al. 2018; Myezwa et al. 2018). This underscores the importance of tailoring interventions aimed at improving adherence and HRQOL to address such factors among PWH in South Africa. Rehabilitation specialists might be particularly well-equipped to encourage cART adherence and mitigate subsequent complications. This is especially pertinent as some of the factors associated with non-adherence could be modified through rehabilitation efforts (Cobbing et al. 2013).

In our sample, a lower (but not the lowest) household income was associated with poorer HRQOL. Positive linear relationships have commonly been established between income and HRQOL in PWH. However, relationships between income gradient and HRQOL – particularly mental domains – have demonstrated to be more complex and may, for example, be negative or non-linear; especially in low-resource or inequal settings (Dageid & Grønlie 2015; Pozniak 2014; Tsevat et al. 2009; Zhang & Xiang 2019). Despite being classified as a high-middle income country, most of South Africa’s population faces excessive levels of inequality and continues to live in poverty-stricken areas with inadequate resources and service delivery (Gordon, Booysen & Mbonigaba 2020). Factors that may mediate the income-HRQOL relationship in such settings include access to healthcare (which may be better in PWH on cART [Pozniak 2014; Tsevat et al. 2009]) and other inequalities in healthcare (Zhang & Xiang 2019), social networking (which is linked to social class inequality in health and may be helpful or detrimental (Zhang & Xiang 2019), and social capital (which may have a particularly strong association with self-rated health in inequal settings [Dageid & Grønlie 2015]). Our study underscores the negative consequences of continuing healthcare inequalities in a country such as South Africa, where one in every five persons aged as young as 15 years reports poor subjective health (Biney, Amoateng & Ewemooje 2020).

We further observed a positive association between HRQOL and objective lower limb function. This, along with almost a fifth of participants reporting mobility problems, underscores the role that rehabilitation specialists can play in optimising patient-important outcomes in HIV care. Relationships between physical function and HRQOL in HIV have recently been demonstrated in relatively older cohorts on ART in a HIC (Erlandson et al. 2014), in ART-naïve PWH in an upper-middle income country (Lédo et al. 2018) and, using self-reported measures, in PWH with distal sensory polyneuropathy (Galantino et al. 2014). Our findings replicated this association in PWH mostly on cART, without PN, and residing in a LMIC. The interplay between HRQOL and PN is intricate and may mediate or confound each other (Riandini et al. 2018). By excluding PN from our analysis, we provide evidence of a direct association of locomotor function with HRQOL in our sample. Given the growing concerns surrounding so-called accelerated ageing in PWH and its potential manifestation as functional decline at earlier-than-expected ages (Quigley & MacKay-Lyons 2020), monitoring functional performance over time in PWH may be crucial for maintaining or optimising HRQOL.

Additionally, longer HIV duration (> 15 years ago) and polypharmacy were associated with poorer HRQOL. It has been suggested that PWH diagnosed in the earlier years of the epidemic may suffer greater detrimental impacts on HRQOL than those diagnosed more recently (Miners et al. 2014). This may be because of current advances in cART regimes and CD4-related initiation criteria (preventing advanced disease), long-term treatment plans and support networks; versus severe treatment side-effects and acquired immunodeficiency syndrome (AIDS)-associated illnesses and disability in earlier years (Miners et al. 2014; Pozniak 2014). Longitudinal HRQOL studies in PWH have also suggested a two-phased course, where HRQOL improves within the first year after cART initiation, followed by a relative stabilisation (Protopopescu et al. 2007). Polypharmacy has also previously demonstrated associations with poorer HRQOL and cART non-adherence, in PWH; independent of existing comorbidities (Okoli et al. 2020) (similar to our findings). The high pill burden, potential drug–drug interactions, and poor clinical outcomes associated with polypharmacy (including falls, delirium, pneumonia, hospitalisation, and death [Edelman, Rentsch & Justice 2020]) likely contribute to these relationships. Polypharmacy is common among PWH, occurring about a decade earlier than in the general population (Edelman et al. 2020). Incidentally, polypharmacy in PWH has been associated with a longer HIV duration, rather than older age (Guaraldi et al. 2018). Although more common in more affluent settings, emerging evidence indicates that the phenomenon also applies to LMICs (Ssonko et al. 2018). Multidisciplinary coordinated models of care, including the incorporation of rehabilitation to enhance health promotion with ageing, are increasingly advocated in the care of complex chronic conditions such as HIV and concurrent multimorbidity (Edelman et al. 2020; O’Brien et al. 2020a). Promoting healthy behaviours such as physical activity may, for example, help in preventing or managing conditions associated with polypharmacy (Edelman et al. 2020); as such potentially contributing to better HRQOL.

Our findings contribute to a growing body of evidence supporting the value of rehabilitation to optimise the management of chronic HIV (O’Brien et al. 2020a). Despite seemingly high HRQOL, PWH in our sample continued to face functional problems and activity limitations and other factors related to self-perceived health in the face of potential health inequalities. The value of comprehensive management is supported by the consideration that the problematic HRQOL domains and factors associated with overall HRQOL may at least partly be amenable to a multidisciplinary approach, including mental health and physical rehabilitation and health promotion. This highlights potential areas of targeted screening, and intervention, that need further understanding in terms of how PWH’s HRQOL can be optimised.

Our findings point to factors that could be considered for monitoring HRQOL in the clinical setting, or that may be linked to evidence-based interventions (e.g., such as exercise) to improve functioning and health to optimise subjective health and subsequently HRQOL, now or in future.

Many of our findings support the existing and emerging literature in the cART era. Reporting of our study adhered to most criteria for methodological and conceptual rigour in QOL studies, as proposed by Gill and Feinstein (1994)(Haraldstad et al. 2019), with the exception of criteria related to patient-supplemented elaborations on questionnaire items, and indications of items that were of personal relevance to respondents. In addition, credibility of the results, and comparability to other studies, are increased using a standardised HRQOL tool available in local languages and that has been validated for South African PWH.

Study limitations include the cross-sectional design, which precludes making causal inferences. The small sample size may increase Type II error, and pose potential challenges of overfitting and model instability in multivariable regression analyses. However, to strike a balance between including meaningful predictors and maintaining model simplicity within the constraints of our dataset, we included a limited number of predictor variables in our final multivariable models (four and three, respectively, for the sample of 48). Our study provides insights into an apparently non-impaired sample from two primary care clinics in a single district of the Western Cape (specifically, non-elderly non-obese PWH without PN). As such, findings are not necessarily generalisable to other clinical settings and may not be considered representative of all South African PWH. Furthermore, HRQOL is a complex and highly subjective phenomenon and the EQ-5D-5L does not provide a comprehensive evaluation of all HRQOL dimensions (e.g., social and spiritual domains). In addition, the quantitative nature of the tool fails to provide a full picture of the complex lived experiences of PWH. Finally, EQ-index scores were calculated using Zimbabwean valuations (weightings), as no South African values were available at the time of writing. It is worth observing that weightings in South Africa could vary because of the different context and attitudes towards health status (Louwagie et al. 2007).

## Conclusion

Our study reveals that, while PWH generally scored high on HRQOL measures, specific challenges remain in domains such as pain or discomfort, depression or anxiety, and mobility. Factors associated with better HRQOL included cART adherence, while slow chair rise, lower income, longer HIV duration, and polypharmacy were associated with poorer HRQOL. The findings suggest that, despite apparently high HRQOL, there are significant challenges that could impact PWH – who are currently relatively young and unimpaired – now and in the long term. Clinicians, particularly at primary care level and including rehabilitation specialists, should be cognisant of such factors that may be considered for monitoring and/or maintaining HRQOL in PWH, or that may be linked to evidence-based interventions. Comprehensive care and contextualised interventions to address the factors related to HRQOL through rehabilitation, including health promotion, are proposed strategies for future investigation via larger-scale, longitudinal studies.
